# Towards the systematic discovery of signal transduction networks using phosphorylation dynamics data

**DOI:** 10.1186/1471-2105-11-232

**Published:** 2010-05-07

**Authors:** Haruna Imamura, Nozomu Yachie, Rintaro Saito, Yasushi Ishihama, Masaru Tomita

**Affiliations:** 1 Institute for Advanced Biosciences, Keio University, Tsuruoka, Yamagata, Japan; 2 Systems Biology Program, Graduate School of Media and Governance, Keio University, Fujisawa, Kanagawa, Japan; 3 Systems Biology Program, Faculty of Environment and Information Studies, Keio University, Fujisawa, Kanagawa, Japan; 4 PRESTO, Japan Science and Technology Agency, Chiyoda-ku, Tokyo, Japan; 5 Current Address: Department of Biological Chemistry and Molecular Pharmacology, Harvard Medical School, Boston, MA 02115, USA

## Abstract

**Background:**

Phosphorylation is a ubiquitous and fundamental regulatory mechanism that controls signal transduction in living cells. The number of identified phosphoproteins and their phosphosites is rapidly increasing as a result of recent mass spectrometry-based approaches.

**Results:**

We analyzed time-course phosphoproteome data obtained previously by liquid chromatography mass spectrometry with the stable isotope labeling using amino acids in cell culture (SILAC) method. This provides the relative phosphorylation activities of digested peptides at each of five time points after stimulating HeLa cells with epidermal growth factor (EGF). We initially calculated the correlations between the phosphorylation dynamics patterns of every pair of peptides and connected the strongly correlated pairs to construct a network. We found that peptides extracted from the same intracellular fraction (nucleus vs. cytoplasm) tended to be close together within this phosphorylation dynamics-based network. The network was then analyzed using graph theory and compared with five known signal-transduction pathways. The dynamics-based network was correlated with known signaling pathways in the NetPath and Phospho.ELM databases, and especially with the EGF receptor (EGFR) signaling pathway. Although the phosphorylation patterns of many proteins were drastically changed by the EGF stimulation, our results suggest that only EGFR signaling transduction was both strongly activated and precisely controlled.

**Conclusions:**

The construction of a phosphorylation dynamics-based network provides a useful overview of condition-specific intracellular signal transduction using quantitative time-course phosphoproteome data under specific experimental conditions. Detailed prediction of signal transduction based on phosphoproteome dynamics remains challenging. However, since the phosphorylation profiles of kinase-substrate pairs on the specific pathway were localized in the dynamics-based network, our method will be a complementary strategy to explore new components of protein signaling pathways in combination with previous methods (including software) of predicting direct kinase-substrate relationships.

## Background

Post-translational modification (PTM) of proteins regulates many biological phenomena [1]. Among the several kinds of PTM, phosphorylation affects enzymatic activity, conformations, interactions, degradation, and localization of proteins, among other effects [2-4]; one of the critical roles of phosphorylation is in the control of protein signaling [5]. More than 500 protein kinases are thought to regulate protein signaling in humans [6]. In protein signaling, various reaction cascades transmit and amplify signals in a highly regulated manner by means of reversible site-specific protein phosphorylation [5]. Kinases recognize the specific surrounding sequences of phosphosites when they phosphorylate their targets, and the majority of the identified kinases are thought to have their own unique target sequences, which are known as "motif sequences" [7].

Liquid chromatography coupled with tandem mass spectrometry (LC-MS/MS), combined with phosphopeptide enrichment technology [8], is a powerful method for identifying large numbers of phosphosites [9]. This technology has achieved phosphorylation analysis at the proteome level and has greatly expanded the new field of phosphoproteomics. In recent years, phosphoproteome data has rapidly increased for various organisms, including humans [10-12], mice [13], yeast [14-16], flies [17,18], plants [19], and bacteria [20,21], as a result of system-wide experiments to investigate the behavior of signal transduction pathways under various stimuli and environmental conditions. For example, at least one-third of all human proteins have been revealed to be phosphorylated [22]. The phosphoproteins and their phosphosites identified in these studies have been stored in public databases, such as Phospho.ELM [23], PHOSIDA [24], PhosphoSitePlus [25], and UniProt [26].

"Phosphoinformatics" approaches (i.e., bioinformatics of the phosphoproteome) have derived many useful biological interpretations from the huge and complex body of phosphoproteome data and have aided in the discovery of novel biological principles of protein signaling [27,28]. Understanding of the relationship between protein kinases and the specific sequence patterns of their phosphorylation targets has increased rapidly and considerable data has accumulated as a result of recent phosphoproteome data mining studies [29,30] or the use of predictive software such as Scansite [31] and NetPhos [32]. Many kinases and their corresponding substrate recognition motifs have been accumulated in databases, such as the Human Protein Reference Database (HPRD) [33], PhosphoSitePlus [25], and NetPhorest [34].

The relationships between enzymes and their substrate motifs are useful for the discovery and reconstruction of signal transduction networks in living systems. Several computational approaches, including NetworKIN [35], have been developed to predict signaling pathways based on the unique phosphorylation target motifs of kinases and other -omics datasets (e.g., protein-protein interaction data and functional relationship data for genes). However, the kinase specificity for target motifs appears to be limited, and therefore the accurate prediction of enzyme-substrate pairs based on the documented motifs of the kinase targets remains difficult. Although the current LC-MS/MS-based phosphoproteome approach excels at the identification of phosphorylated components of the proteome, the identification of kinase-substrate relationships is fraught with challenges [36].

On the other hand, by taking advantage of protein labeling methods, the high-throughput LC-MS/MS-based proteomics approaches have enabled us to compare different states of intracellular proteomes, and several studies have revealed the time course of phosphoproteome behaviors [11,37,38]. Many phosphoproteins have been shown to have multiple potential phosphosites [28], and the phosphosites in each protein behaved in different ways in response to different external stimuli. The time-course profile of phosphorylation states at individual phosphosites across multiple cellular states can be used to capture intracellular signal transitions, for example, where signals are propagated from a receptor on the cell membrane to downstream pathways [39]. Recently, several enzyme-substrate prediction approaches have started to use such time-course phosphoproteome data to solve the challenge of accurately predicting signal transduction pathways [40]. Since the phosphorylation profiles of substrates are affected by their corresponding kinases, most of whose enzymatic activities are enhanced by phosphorylation, it has been generally assumed that in many cases, a kinase and its substrate have similar phosphorylation profiles. This concept of a "projection effect" of signaling proteins seems to work well to predict direct relationships between a kinase and its substrates in signaling pathways based on quantitative phosphoproteome data. For example, Jørgensen and colleagues [27] have reported the combination of quantitative phosphoproteome data with computational prediction of the signaling pathway utilizing NetworKIN and NetPhorest, and Locasale and Wolf-Yadlin [40] have reported a new approach for the prediction of a tyrosine signaling pathway in which they used the principle of maximum entropy to represent similarities in the phosphorylation profile as a network construct and to predict specific pathways.

In this paper, we propose a phosphorylation dynamics-based network approach that roughly clusters the proteins localized within a condition-specific signaling pathway. Although the maximum entropy-based approach [40] predicts pair-wise connections of proteins in tyrosine signaling, our proposed approach clusters all types of phosphoproteins (pS, pT, and pY proteins) in a network graph and suggests proteins that are localized within a specific pathway activated by a given experimental condition under which the dynamics of the phosphoproteome are measured. In this study, we used the time-course phosphoproteome data for the epidermal growth factor (EGF) stimulation of HeLa cells previously obtained using LC-MS/MS based on the stable isotope labeling using amino acids in cell culture (SILAC) approach [11]. This phosphoproteome dynamics data consists of the phosphorylation activities of digested phosphopeptides at five time points after the EGF stimulation. Using this data, we constructed a dynamics-based network by connecting pairs of phosphopeptides that exhibited similar patterns of phosphorylation dynamics. According to several analyses based on graph theory and comparisons with known signaling pathways registered in public databases, we demonstrated that the phosphopeptides corresponding to proteins that participated in a signaling pathway were clustered within the dynamics-based network. Moreover, we found that the dynamics-based network for EGF stimulation was more strongly correlated with the known EGF receptor (EGFR) signaling pathway than with other known pathways. This suggests the biological specificity of the EGFR signaling pathway. Although the time-course data show that many phosphorylation sites, including those that are not thought to be involved in EGFR signaling, seemed to be activated by the EGF stimulation, only the known EGFR signaling pathway was markedly correlated with the time-course data at the pathway level. The EGFR signaling pathway thus seemed to be precisely controlled to avoid activation of unrelated pathways. The phosphorylation dynamics-based network approach will therefore prove to be a useful strategy for providing an overview of and for exploring condition-specific protein signaling; although it cannot replace direct prediction methods, it can help to improve their accuracy.

## Results and Discussion

### Construction of the phosphorylation dynamics-based network

The projection effect exists in protein signaling: some phosphorylation reactions are known to transfer signals from one molecule to another (i.e., project their effects onto the other molecule), and these reactions occur continuously (e.g., when kinase A phosphorylates protein B, then protein B is activated and starts to behave as a kinase; protein B, in turn, phosphorylates its target substrates). Because most kinases are thought to gain their enzymatic activities as a result of phosphorylation, these signaling proteins and their targeted substrates should have similar time courses for their phosphorylation behaviors. In this context, we proposed using a phosphorylation dynamics-based network approach to cluster proteins localized within a signaling pathway.

In this study, we used the time-course phosphoproteome data previously reported by Olsen *et al*. [11]. This data provides the relative phosphorylation activities of digested peptides at each of five time points (0, 1, 5, 10, and 20 min) after the EGF stimulation (for details, see the Methods section). We extracted 1,050 peptides for which the relative phosphorylation activities were available at each of the five time points. Using this time-course data, we constructed dynamics-based networks by connecting all pairs of phosphopeptides with similar phosphorylation activity profiles. We calculated the similarity of every possible pair of phosphorylation profiles using Pearson's correlation coefficient (*R*). The dynamics-based network was then constructed to connect all phosphopeptide pairs with *R *values greater than a given threshold.

**Figure 1:**
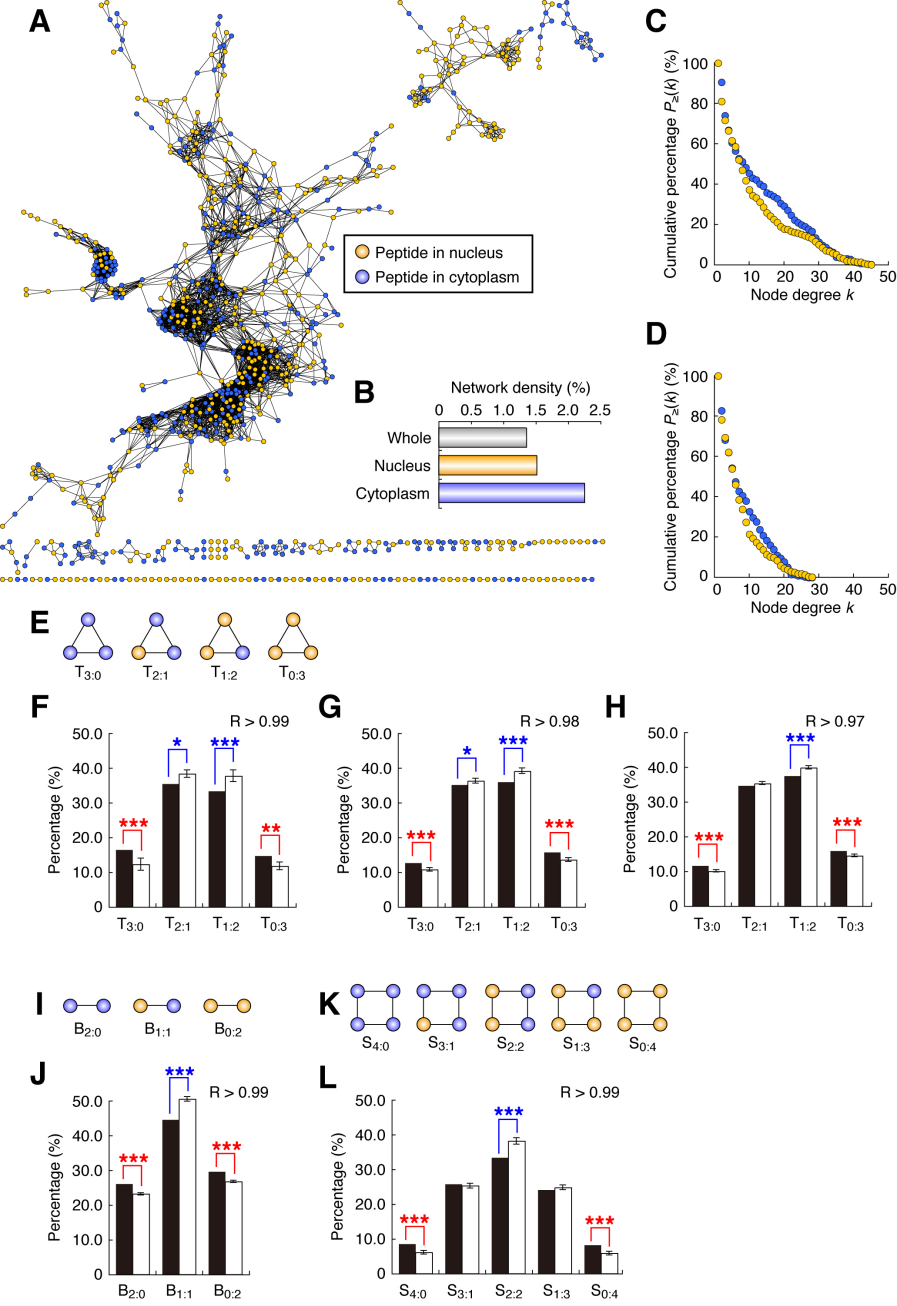
**Characteristics of the phosphorylation dynamics-based network. **(**A**) We generated the dynamics-based network by connecting pairs of peptides with similar (*R *> 0.99) time courses of phosphorylation activities. The network was visualized using Cytoscape (version 2.6.1) [45] and eXpanda (version 1.0.6) [46]. (**B**) Network density of the whole dynamics-based network and of the cytoplasmic and nucleic subnetworks. (**C**) Cumulative proportion, *P *≥ (*k*), for the node degrees (*k*) based on analysis of the whole dynamics-based network simultaneously (i.e., not separately as in Figure D) (*R *> 0.99). For each group of cytoplasmic and nucleic nodes in the network, circles represent the proportions of proteins having more than *k *interacting partners. (**D**) Cumulative proportion for the node degrees with the cytoplasmic and nucleic subnetworks analyzed separately. (**E, I, K**) Patterns of the cellular fractions (cytoplasmic and nucleic): (**E**) binary, (**I**) triangular, and (**K**) square motifs that appeared in the dynamics-based network. The names of each motif pattern appear under the corresponding diagram: T, triangular; B, binary; S, square. (**F-H, J, L**) Appearance of each motif (proportion of total) in the dynamics-based network. (**F-H**) Triangular motifs appeared in the dynamics-based network of (**F**) *R *> 0.99, (**G**) *R *> 0.98, and (**H**) *R *> 0.97. (**J**) Binary and (**L**) square motifs appeared in the dynamics-based network with *R *> 0.99. Black bars represent percentages of the corresponding motif patterns in the real dynamics-based network; white bars represent the mean values of the percentages estimated using negative controls generated by random edge rewiring (RER, *n *= 1000). Error bars represent standard deviations. Significance levels: *, *P *< 0.05; **, *P *< 0.01; ***, *P *< 0.001.

We constructed three dynamics-based networks, using threshold values of *R *> 0.97, 0.98, and 0.99. The dynamics-based network reconstructed with a threshold value of *R *> 0.99 contained 4,907 edges and 851 nodes (of which 377 and 474 nodes were cytoplasmic and nucleic, respectively; Figure 1A and Additional File 1). The network with *R *> 0.98 consisted of 10,626 edges and 959 nodes (of which 423 and 536 were from the cytoplasm and nucleus, respectively; Additional File 2), and the network with *R *> 0.97 consisted of 16,481 edges and 1,015 nodes (of which 442 and 573 were from the cytoplasm and nucleus, respectively; Additional File 3).

### EGFR signaling pathways are more abundant in the cytoplasm than in the nucleus

To determine whether the phosphorylation dynamics-based network reflected the biological properties of a cell, we analyzed the network features using graph theory. First, we separated the dynamics-based network with *R *> 0.99 into cytoplasmic and nucleic subnetworks (for details, see the Methods section). The network densities of the cytoplasmic and nucleic networks were 2.25% and 1.52%, respectively (Figure 1B). The higher density in the cytoplasmic subnetwork indicates that in the cytoplasm, more peptides have similar phosphorylation patterns than in the nucleus, which in turn suggests that the EGF stimulation triggers more phosphorylation reactions in the cytoplasm than in the nucleus.

Consistent with this observation, the overall degrees of the cytoplasmic nodes were higher than those of the nucleic nodes in the dynamics-based network with *R *> 0.99 (Figure 1C), although fewer phosphopeptides were identified in the cytoplasm than in nucleus, as described previously (in the section "Construction of the phosphorylation dynamics-based network"). We obtained similar results even if we calculated node degrees separately in subnetworks that consisted only of nucleic nodes and only of cytoplasmic nodes (Figure 1D). These results may reflect the fact that in the larger cellular space of the cytoplasm, many long and alternative pathways can be activated to amplify and transmit signals into the nucleus, where the mandatory signals are transmitted through shorter, more highly regulated nuclear pathways to precisely control gene expression.

### Time course of the phosphoproteome reflects the cellular fraction

The network density for the whole dynamics-based network with *R *> 0.99 (1.36%) was sparser than the densities of the cytoplasmic and nucleic subnetworks (Figure 1B). As illustrated in Figure 1A, this suggests that in the dynamics-based network, phosphopeptides from the same cellular fraction (i.e., the cytoplasm or nucleus) are tightly connected, whereas connections are less likely between the cytoplasmic and nucleic nodes.

We analyzed the patterns of several local network motifs that appeared in the dynamics-based network, where each node was either nucleic or cytoplasmic (Figure 1E, I, and 1K). The triangular motif (in which the three nodes are connected together by three edges) in a biological network is the smallest clump of nodes that reveals the connection patterns of nodes in a locally dense network, and is thought to be a basic motif that reflects biologically relevant phenomena [16]. As Figure 1E shows, there are four possible triangular motifs (T) in the dynamics-based network, which consist of various combinations of nucleic and cytoplasmic nodes: all nodes are cytoplasmic (T_3:0_), two are cytoplasmic and one is nucleic (T_2:1_), one is cytoplasmic and two are nucleic (T_1:2_), and all nodes are nucleic (T_0:3_). In each of the dynamics-based networks (with *R *> 0.97, 0.98, and 0.99), we calculated the proportion of each of the four triangular motifs and compared it with the expected results generated by random edge rewiring (RER, used as the negative control; *n *= 1,000; for details, see the Methods section). In the dynamics-based network with *R *> 0.99, the proportions of T_3:0 _and T_0:3 _were 16.50% and 14.75%, respectively, which were significantly higher than the expected negative control values of 12.24 ± 1.74% (s.d.) and 11.77 ± 1.12% in the random networks (*P *< 0.001 and 0.01, respectively; Figure 1F). Conversely, the proportions of T_2:1 _and T_1:2 _were 35.40% and 33.34%, which were significantly lower than the corresponding control values of 38.30 ± 1.09% and 37.69 ± 1.70% (*P *< 0.05 and 0.001, respectively; Figure 1F). The dynamics-based networks generated with thresholds of *R *> 0.97 and 0.98 showed similar patterns (Figure 1G, H). Consequently, the triangular motif analysis supports the hypothesis that phosphopeptides identified in the same cellular fraction were localized (clustered) in the dynamics-based network. We also analyzed the number of binary (Figure 1I) and square (Figure 1K) motifs in the dynamics-based network with *R *> 0.99, and observed similar patterns (Figure 1J, L).

The connectivity of phosphopeptides within a given cellular fraction suggests that the phosphorylation dynamics-based network is a valid approach for the reconstruction of the entire protein-based signal pathway, because in most cases, signal transmission occurs between proteins that lie close together. We expected that the validity of our approach would depend partly on the threshold *R*. To test this hypothesis, we defined a phosphopeptide localization score for the dynamics-based network as the sum of the proportions T_3:0 _and T_0:3_, and calculated the *O*/*E *value for the localization score (where *O *is the observed localization score in the real network and *E *is the expected average score in the RER network with *n *= 1,000). The resulting average *O*/*E *values with *R *> 0.99, 0.98 and 0.97 were 1.30, 1.17, and 1.12, respectively. Therefore, the higher *R *threshold generates a dynamics-based network that more accurately reflects the actual intracellular signaling network.

### The EGF stimulation activates many signaling proteins

Given the projection effect of protein signaling, proteins whose phosphorylation behaviors are similar to those of many other proteins are thought to be activated by upstream signals and to transmit a signal to downstream proteins. Accordingly, in the dynamics-based network for the EGF stimulation, phosphopeptides with many interacting partners (a high node degree) would have corresponding proteins that are activated by the EGF stimulation. Indeed, many biological function annotations (derived by means of gene ontology) of the phosphopeptide nodes represented as hubs in the dynamics-based network with *R *> 0.99 were related to signal transmission (e.g., "cell cycle" and "insulin receptor signaling"; Table 1) and *vice versa *(Additional File 4). These results suggest that many signaling proteins, including those unrelated to the EGFR signaling, gain their enzymatic activities as a result of the EGF stimulation (see the section "The EGFR signal is precisely transmitted to its downstream components under the control of whole-proteome pathways" for additional discussion of this point).

**Table 1:** Proteins with a degree ≥ 30 in the dynamics-based network.

**IPI accession**	**Degree**	**Biological process**
IPI00002591	45	phosphatidylinositol biosynthetic process; receptor-mediated endocytosis; phosphoinositide phosphorylation; phosphoinositide-mediated signaling
IPI00294391	44	regulation of cell shape; signal transduction
IPI00438229	43	epithelial to mesenchymal transition; regulation of transcription from RNA polymerase II promoter; positive regulation of gene-specific transcription
IPI00148057	42	cell surface receptor linked signal transduction; chromatin modification; regulation of transcription from RNA polymerase II promoter; regulation of growth
IPI00291916	42	insulin receptor signaling pathway
IPI00000858	40	regulation of transcription, DNA-dependent
IPI00299263	40	intracellular protein transport; protein secretion; vesicle-mediated transport; regulation of ARF GTPase activity
IPI00093253	39	double-strand break repair via homologous recombination
IPI00009975	39	regulation of transcription, DNA-dependent
IPI00003406	38	regulation of dendrite development; actin filament organization; regulation of neuronal synaptic plasticity
IPI00170770	37	multicellular organismal development; transcription
IPI00550206	37	regulation of transcription, DNA-dependent
IPI00017030	37	anti-apoptosis
IPI00464952	36	nuclear mRNA splicing, via spliceosome
IPI00010141	36	DNA replication
IPI00552897	36	DNA repair; cell cycle
IPI00026673	35	multicellular organismal development; negative regulation of transcription from RNA polymerase II promoter
IPI00298731	35	protein import into nucleus; transcription
IPI00425404	35	microtubule-based movement
IPI00217957	34	multicellular organismal development; transcription
IPI00438170	34	protein transport; cell communication
IPI00097495	34	regulation of Rab GTPase activity
IPI00021954	33	retrograde vesicle-mediated transport, Golgi to ER; COPI coating of Golgi vesicle; regulation of ARF protein signal transduction
IPI00298935	33	oxidation reduction; chromatin modification; regulation of transcription, DNA-dependent
IPI00414262	32	activation of JUN kinase activity
IPI00181006	32	regulation of transcription, DNA-dependent; apoptosis
IPI00004472	32	protein amino acid phosphorylation; protein kinase cascade; ion transport
IPI00431698	32	interspecies interaction between organisms; regulation of transcription, DNA-dependent
IPI00004233	32	cell proliferation; cell cycle
IPI00296388	31.33	regulation of transcription, DNA-dependent; chromatin remodeling
IPI00219430	30	transcription initiation from RNA polymerase II promoter; androgen receptor signaling pathway; regulation of transcription, DNA-dependent
IPI00301503	30	RNA splicing, via transesterification reactions; nuclear mRNA splicing, via spliceosome

Here, "Node degree" indicates the mean degree of phosphopeptides of the corresponding protein in the dynamics-based network with *R *> 0.99. The biological process represents the result annotated by means of gene ontology, as described in the Methods section.

### The phosphorylation dynamics-based network reflects known signaling pathways

To evaluate whether the dynamics-based network reflects actual signaling phenomena at the pathway level, we compared the network with *R *> 0.99 with known signal transduction pathways registered in public databases.

For this comparison, we used the shortest path length (SPL; for details, see the Methods section) to evaluate the closeness of pairs of nodes in the network and the pathways. In the dynamics-based network, each edge between two nodes denotes a similarity in the time course of the phosphorylation profiles, and in the known signaling pathways, each edge that connects an enzymatic protein and its substrate represents a phosphorylation reaction. Although the edges in the known signaling pathways are directional, we treated them as nondirectional in this study because we only needed to investigate whether the phosphorylation dynamics patterns of two phosphosites were strongly correlated when their corresponding proteins were located close together in the known pathway, regardless of the reaction direction.

**Figure 2:**
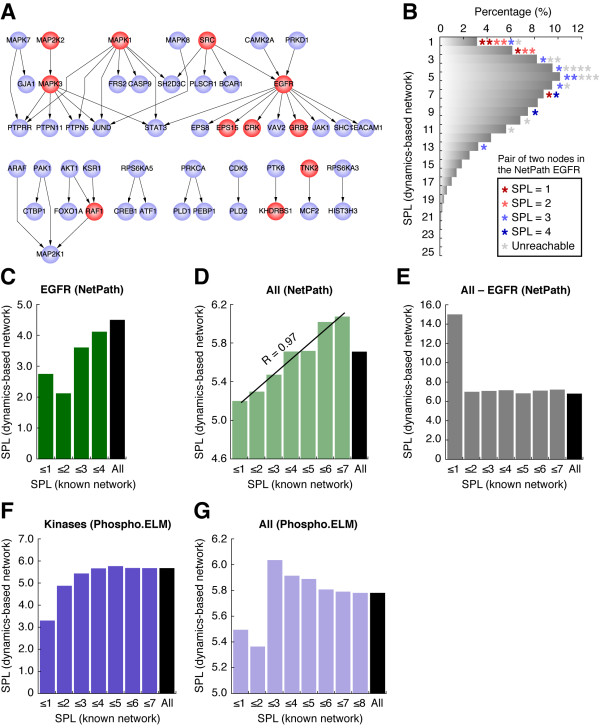
**Comparison of the dynamics-based network with known cellular signaling pathways. **(**A**) Known epidermal growth factor receptor (EGFR) signal transduction pathways in the EGFR (NetPath) analysis obtained from the NetPath database. Phosphorylation reactions are indicated by directed arrows from protein kinases to their target substrate. Red protein nodes indicate that the phosphorylation dynamics of the peptide or peptides were included in the dynamics-based network with *R *> 0.99. (**B**) Density distribution for the shortest path lengths (SPLs) of all reachable two-phosphopeptide nodes in the dynamics-based network (bars). Each asterisk denotes a pair of proteins in the EGFR (NetPath) known signaling pathway data; the asterisk color denotes the SPL of the two proteins in the known signaling network, and the position of each asterisk denotes the SPL of the corresponding two phosphopeptides in the dynamics-based network. (**C-G**) Comparison of SPLs in the dynamics-based network with those in each of the five known signaling networks. (**C**) EGFR (NetPath), (**D**) All (NetPath), (**E**) All - EGFR (NetPath), (**F**) Kinases (Phospho.ELM), and (**G**) All (Phospho.ELM). In each panel, the SPLs of the two proteins in the known network were assigned to the bins indicated on the horizontal axis; for protein pairs in each bin, we calculated the SPLs of their corresponding peptide pairs in the dynamics-based network (only for the reachable peptide pairs in this network), and the resulting mean value is shown on the vertical axis. The bin labeled "All" also included unreachable pairs in the known network. (**D**) In the comparison with EGFR (NetPath), Pearson's correlation coefficient (*R*) was calculated without the "All" bin.

We initially obtained 46 kinase-substrate reactions in the EGFR signaling pathway from NetPath [41] as the EGFR (NetPath) pathway (Figure 2A). Since the dynamics-based network in this study was constructed on a pilot scale by connecting only proteins with similar phosphorylation profiles, we did not use phosphatase-related reactions that are believed to have inversely correlated time-course profiles between the phosphatase and its substrate. In the EGFR (NetPath) pathway, 11 proteins (red nodes in Figure 2A) matched to the dynamics-based network; for these 11 proteins, 30 protein pairs had corresponding phosphopeptides that were reachable in the dynamics-based network. We compared the SPLs of these 30 protein pairs in the EGFR (NetPath) pathway with the SPLs of their corresponding phosphopeptide pairs in the dynamics-based network, and found a marked correlation between the two datasets (Figure 2B and 2C), suggesting that the dynamics-based network for the EGF stimulation accurately clustered proteins that are close to each other in the actual EGFR signaling pathway.

The data size for the EGFR (NetPath) pathway was limited. To quantitatively measure the correlation between the dynamics-based network and the known pathways with a larger dataset, we prepared two other datasets of known signaling pathways from NetPath: All (NetPath), consisting of a total of 296 phosphorylation reactions in the database, and All - EGFR (NetPath), generated by eliminating all the EGFR (NetPath) reactions from the All (NetPath) pathways. Although the SPLs of the All (NetPath) group were significantly correlated with those of the dynamics-based network (Figure 2D), the All - EGFR (NetPath) had no correlation with the dynamics-based network (Figure 2E). The average SPLs of the phosphopeptides in the dynamics-based networks that corresponded to protein pairs in the known datasets of EGFR (NetPath), All (NetPath), and All - EGFR (NetPath) were 4.50, 5.71, and 6.80, respectively (Figure 2C-E). Moreover, those SPLs which corresponded to neighboring protein pairs (i.e., SPL = 1) in the same datasets were 2.75, 5.20, and 15.00, respectively (Figure 2C-E). Thus, two proteins that participated in the EGFR signaling pathway were also shown to be close in the dynamics-based network for the EGF stimulation, and those localized in the non-EGFR signaling pathway were shown to be more distant. The correlation between the dynamics-based network and the All (NetPath) pathway might have resulted from the indirect effect of the proteins belongs to the EGFR signaling pathways in the All (NetPath) group.

We further compared the dynamics-based network with two known signaling pathway networks generated from Phospho.ELM [23]: All (Phospho.ELM), consisting of a total of 1,140 kinase-substrate reactions, and Kinases (Phospho.ELM), generated by selecting 201 protein pairs in which both were annotated as protein kinases from the All (Phospho.ELM) dataset. Since Phospho.ELM covers phosphorylation reactions that were identified by means of large-scale, high-throughput measurements, and might include reactions that are not meaningful in living cells, the Kinases (Phospho.ELM) pathway was the more biologically relevant dataset. We confirmed that the dynamics-based network for the EGF stimulation was more similar to the Kinases (Phospho.ELM) group than to the All (Phospho.ELM) (Figure 2F and 2G); for example, the average SPLs in the dynamics-based network corresponding to neighboring protein pairs with SPL = 1 in the respective datasets of Kinases (Phospho.ELM) and All (Phospho.ELM) were 3.33 and 5.50, respectively. These results suggest that the dynamics-based network constructed according to the time-course data for the EGF stimulation reflects the actual intracellular EGFR signaling pathways, and that our approach can be used to understand proteins localized in other condition-specific signaling pathways.

### The EGFR signal is precisely transmitted to its downstream components under the control of whole-proteome pathways

As discussed above, given the projection effect of protein signaling, the fact that many signaling proteins unrelated to EGFR signaling appear as hubs in the dynamics-based network (Table 1, Additional File 4) indicates that these unrelated signaling proteins may be activated by the EGF stimulation. Many signaling proteins seem to be indiscriminately activated, and this would lead to side-effects that transmit and amplify inappropriate signals within the cell. However, we found that the dynamics-based network was correlated specifically with the EGFR signaling pathway (Figure 2C-E). In particular, the average SPL for the phosphopeptide pairs in the dynamics-based network that corresponded to neighboring protein pairs in the EGFR signaling pathway was 2.75, whereas that in the non-EGFR signaling pathway was 15.00 (Figure 2C and 2E). The adjacent protein pairs in the non-EGFR signaling were therefore farther apart in the dynamics-based network for the EGF stimulation. These results suggest that at the pathway level, the EGFR signaling system is precisely activated to transmit and amplify signals along the appropriate intracellular pathway and to not transmit signals along inappropriate pathways.

## Conclusions

The dynamics-based network generated using the time-course phosphoproteome data for the EGF stimulation was clearly correlated with the known EGFR signaling pathways. Although recently developed computational methods predict direct signaling relationships between enzyme proteins and their substrates based on substrate-recognition motifs, our network construction approach is useful because it groups pairs of proteins localized within a signaling pathway based on the similarity of their phosphorylation dynamics data. The dynamics-based network, in turn, will complement the results of enzyme-substrate predictions based on the enzyme-specific target sequence motif (e.g., NetworKIN, Scansite, and NetPhos) and will therefore increase their prediction accuracy. The new approach will also serve as a guide to explore enzyme targets even when the enzyme's motif information is unavailable. Furthermore, the dynamics-based network for the EGF stimulation allowed us to infer that EGFR signaling is independently activated and precisely controlled at the whole-pathway level under conditions where many unrelated enzymatic proteins are activated by the EGF stimulation. In the near future, when we obtain more condition-specific time-course phosphoproteome data with higher resolution, the phosphorylation dynamics-based network approach will improve both our general understanding of whole cellular signaling pathway and our understanding of conditionally activated signaling pathways; it will not replace recently developed prediction approaches for direct enzyme-substrate relationships, but will improve their accuracy.

## Methods

### Time-course phosphoproteome data

We used the time-course phosphoproteome data of Olsen *et al. *[11] in this study. In this dataset, the relative abundances of cytoplasmic and nucleic phosphopeptides of HeLa cells were measured at five different time points (0, 1, 5, 10, and 20 min) after EGF stimulation using SILAC-based LC-MS/MS analysis. In this dataset, each phosphopeptide had a unique identifier along with the International Protein Index (IPI) [42] accession numbers of the corresponding protein, the relative abundances at each of the five time points, and the cellular fraction in which each was isolated (cytoplasm or nucleus). Note that throughout this study, we defined a given phosphopeptide that was obtained from both cellular fractions (nucleus and cytoplasm) as two different phosphopeptides. We selected 1,050 phosphopeptides whose relative abundances were completely measured at all five time points for additional analysis. Among the 1,050 phosphopeptides, 459 were cytoplasmic and 591 were nucleic. Since some IPI accession numbers in Olsen *et al*. were outdated, we updated these numbers according to a newer version of the human IPI data (version 3.58).

### Phosphorylation dynamics-based network

Using the time-course phosphoproteome data, we generated a phosphorylation dynamics-based network by connecting pairs of phosphopeptide nodes with similar time-course abundance profiles; the degree of similarity was defined using Pearson's correlation coefficient (*R*). Among the time-course phosphoproteome data, *R *values for all possible pairs of phosphopeptides were calculated using the R statistical software (version 2.8.0) [43], and we generated a phosphorylation dynamics-based network by applying a given threshold value of *R*. In this study, we used three *R *thresholds (0.97, 0.98, and 0.99) to generate three corresponding dynamics-based networks.

### Subnetworks of the dynamics-based network

The phosphorylation dynamics-based network with *R *> 0.99 was separated into two subnetworks based on the two cellular fractions: a cytoplasmic subnetwork and a nucleic subnetwork. For example, the cytoplasmic subnetwork was composed of cytoplasmic phosphopeptide nodes and edges connecting pairs of cytoplasmic nodes.

### Node degree and network density

We used the cumulative percentage distribution to provide an overview of the node degrees (the number of connections at each node) in a given dynamics-based network. We also calculated the network density to measure the overall connectivity within a given dynamics-based network. Here, network density represents the proportion of connected edges in a network relative to all possible node pairs. That is:

where *E *and *N *are the numbers of edges and nodes, respectively.

### Negative control of the dynamics-based network

We used random edge rewiring (RER) to prepare negative controls for the dynamics-based network. RER randomly selects two edges within a given network and randomly rewires (connects) them; it repeats this operation a sufficient number of times until all pair-wise interactions in the queried network have disappeared or until the number of iterations reaches 100 times the number of interactions. During this process, each rewiring operation is retried if a pair of nodes redundantly wired at two edges occurs in the network. RER does not change the degree of each node in a given network.

### Network motifs in the dynamics-based network

We observed the cellular fraction patterns (cytoplasmic or nucleic) of the binary, triangular, and square motifs (Figure 1) revealed in the dynamics-based network. For the dynamics-based networks with *R *> 0.97, 0.98, and 0.99, we counted the numbers of these motifs and compared the results with those estimated using the negative controls.

### Gene Ontology

Gene ontology annotations of human proteins were obtained from Swiss-Prot (version 57.1) using the Swiss-Prot IDs [26] (see below).

### Known signaling and kinase-substrate reaction datasets

To validate whether the dynamics-based network reflected actual intracellular phosphorylation reactions, we compared the dynamics-based network for *R *> 0.99 with datasets [23,41] for the following five types of known signaling pathways: From NetPath, we used all the available phosphorylation reactions, only the phosphorylation reactions related to EGFR signaling (the "EGFR1 signaling pathway"), and all non-EGFR signaling reactions to create the known-pathway datasets "All (NetPath)", "EGFR (NetPath)", and "All - EGFR (NetPath)", respectively. From Phospho.ELM (version 8.2), we obtained all available kinase-substrate reactions, and denoted these as the "All (Phospho.ELM)" dataset. Among these pathways, we generated a subset composed only of kinase proteins, denoted the "Kinases (Phospho.ELM)" dataset, based on the kinase information registered in the Human Protein Initiative (Release 57.13) [44].

### Identifier standardization using the Swiss-Prot ID

All protein and peptide identifiers in the multiple datasets used in this study were standardized into Swiss-Prot ID numbers to permit data integration and comparison. Each peptide identifier in the dynamics-based network was assigned to its corresponding Swiss-Prot ID according to the identifier cross-reference list in the IPI database (version 3.58), the Swiss-Prot database (version 57.1), or both. (Note that in some cases, multiple peptides corresponded to a single Swiss-Prot ID.) UniProt accessions of proteins in the documented NetPath and Phospho.ELM phosphorylation reactions were standardized to their corresponding Swiss-Prot IDs according to the cross-reference lists in Swiss-Prot (version 57.1) and UniProt (Release 12), respectively. Since the protein accessions in Phospho.ELM are based on UniProt (Release 12.3), we used the cross-references in UniProt (Release 12), which was the nearest available version, for the Phospho.ELM data.

### Shortest path length (SPL)

We defined the closeness of two nodes in a network using the shortest path length (SPL), which represents the minimum number of steps (edges) between two nodes. The SPL of every possible and reachable pair in a given network was calculated using the Dijkstra algorithm provided by version 1.4 of the Boost::Graph package, which was obtained from the Comprehensive Perl Archive Network (http://www.cpan.org/).

### Statistics

We determined the statistical significance of the differences between a single real value and the expected value from a group of repeatedly generated random values by calculating the proportion of random values that were greater than or equal to the real value (or less, depend on the instances).

## Authors' contributions

HI performed all computational analyses. NY designed the study and helped with the computational analyses. RS supervised the research and helped to interpret the data. YI and MT supervised the study. All authors read and approved the final manuscript.

## Supplementary Material

Additional file 1 The dynamics-based network reconstructed for *R *> 0.99. Click here for file

Additional file 2 The dynamics-based network reconstructed for *R *> 0.98. Click here for file

Additional file 3 The dynamics-based network reconstructed for *R *> 0.97. Click here for file

Additional file 4 Node degrees in the dynamics-based network for *R *> 0.99 and the corresponding biological processes from the gene ontology annotation. Click here for file
